# LINC00323 knockdown suppresses the proliferation, migration, and vascular mimicry of non-small cell lung cancer cells by promoting ubiquitinated degradation of AKAP1

**DOI:** 10.1016/j.ncrna.2024.12.006

**Published:** 2024-12-14

**Authors:** Bin Ke, Hai Zhong, Yuxin Gong, Xiaofei Chen, Chenxin Yan, Lin Shi

**Affiliations:** a Department of VIP Ward, State Key Laboratory of Oncology in South China, Guangdong Provincial Clinical Research Center for Cancer, Sun Yat-sen University Cancer Center, Guangzhou, 510060, Guangdong, China; b Department of Thoracic Surgery, Zhujiang Hospital of Southern Medical University, Guangzhou, 510282, Guangdong, China; c Department of Respiratory Diseases, Zhujiang Hospital of Southern Medical University, Guangzhou, 510282, Guangdong, China; d Zhujiang Hospital of Southern Medical University, Guangzhou, 510282, Guangdong, China; e Department of Traditional Chinese Medicine, Guangdong Provincial Key Laboratory of Malignant Tumor Epigenetics and Gene Regulation, Guangdong-Hong Kong Joint Laboratory for RNA Medicine, Sun Yat-Sen Memorial Hospital, Sun Yat-Sen University, Guangzhou, 510120, Guangdong, China

**Keywords:** Non-small cell lung cancer, LINC00323, Vascular mimicry, AKAP1, Ubiquitination

## Abstract

**Background:**

LINC00323, a new long noncoding RNA, is aberrantly expressed in several cancers. However, the expression, function, and mechanism of LINC00323 in non-small cell lung cancer (NSCLC) are unclear.

**Methods:**

In the present study, LINC00323, VEGFA, microvessel density (MVD), and AKAP1 levels were confirmed in NSCLC tissues. Cell proliferation, migration, and vascular mimicry (VM) were examined to assess the effects of LINC00323 and AKAP1 on NSCLC cells. In addition, the interaction between LINC00323 and AKAP1 was verified by RNA pull-down, LC-MS/MS and RNA immunoprecipitation. The ubiquitination level of AKAP1 was also confirmed through coimmunoprecipitation, cycloheximide (CHX) chase, and ubiquitination assays in vitro.

**Results:**

Our results revealed that LINC00323 was upregulated in NSCLC tissues and was positively correlated with metastasis, poor prognosis, VEGFA expression, elevated MVD, and AKAP1 expression. Functionally, LINC00323 or AKAP1 knockdown suppressed the proliferation, migration, and VM of NSCLC cells. Mechanistically, LINC00323 could target AKAP1, and LINC00323 knockdown accelerated ubiquitination-mediated AKAP1 protein degradation. Moreover, LINC00323 silencing suppressed NSCLC cell progression by downregulating AKAP1.

**Conclusions:**

LINC00323 knockdown prevents NSCLC cell proliferation, migration, and VM formation by targeting AKAP1, indicating that LINC00323 and AKAP1 might be biological targets for NSCLC treatment.

## 1 Introduction

There were approximately 2.21 million new cases of lung cancer globally in 2020, ranking second among diagnosed cancers, with more than 1.8 million deaths [1]. Non-small cell lung cancer (NSCLC) is the primary type of lung cancer, accounting for 80%–85 % of all lung cancer cases [2]. The development of NSCLC is associated with chronic lung infections, air pollution, long-term smoking, chemical exposure, etc. [3]. The 5-year survival rate of NSCLC patients is very low because of the side effects of surgery, radiotherapy, chemotherapy, and drug resistance [4]. Therefore, it is crucial to discover new drug mechanisms on the basis of existing treatments.

High proliferation and invasion rates are the leading causes of high mortality in NSCLC patients [5]. Vascular mimicry (VM), a new mode of tumor angiogenesis in highly aggressive tumors [6], plays a crucial role in multiple cancer processes, including NSCLC [[7], [8], [9]]. VM is a unique duct-like structure formed by self-deformation and stromal remodeling of tumor cells containing red blood cells [10]. The unique histological structure of VM makes it easier for tumor cells to metastasize through the blood, and detached tumor cells are more likely to metastasize remotely to the bloodstream [11]. Only tumor cells with high malignancy, low differentiation, and strong plasticity can form VM [12]. Therefore, antivascular therapy has become a critical tool in cancer treatment. Drugs targeting angiogenic factors have long been applied in clinical practice to treat various malignancies, including NSCLC [13,14]. However, the antitumor effect of existing antiangiogenic drugs is poor in most patients, and the overall survival of most patients with malignancies is not significantly prolonged [15]. Therefore, further exploration of target genes that can act on VM in cancer cells is essential for NSCLC therapy.

Long noncoding RNAs (lncRNAs) are noncoding protein transcripts [16]. In the NSCLC process, aberrantly expressed lncRNAs can regulate gene signaling networks and alter cell proliferation, metastasis, apoptosis, and chemoresistance [[17], [18], [19]]. Several studies have also shown that lncRNAs can affect VM in multiple types of cancer cells, such as pancreatic cancer [20], renal cell carcinoma [21], glioma [22], and gastric cancer [23] cells. On the basis of the GSE10245 dataset, LINC00323, a new lncRNA, was reported to be highly expressed in NSCLC [24]. In addition, research has shown that LINC00323 is upregulated and may be a prognostic factor for gastric cancer patients [25]. However, the role and mechanism of LINC00323 in VM formation in NSCLC cells are unclear.

Therefore, this research focused mainly on the relationship between LINC00323 and VM in NSCLC cells and related mechanisms, which might provide a new direction for NSCLC therapy.

## 2 Materials and methods

### 2.1 Clinical specimens

Tumor tissues and normal tissues (>3 cm from the tumor margin) were collected from NSCLC patients who underwent surgical treatment between March 2016 and December 2018 at Zhujiang Hospital of Southern Medical University Hospital. Not all patients had been treated with radiotherapy, chemotherapy, immunotherapy, or targeted drugs. Histopathological diagnoses were determined by 2 or more experienced pathologists. The study was approved by the Ethics Committee of Zhujiang Hospital of Southern Medical University (Approval number: 2024-KY-105-01). All the subjects signed an informed consent form.

### 2.2 Cell culture

A549 (SCSP-503) and H1650 (SCSP-592) cells were obtained from the National Collection of Authenticated Cell Cultures (Shanghai, China). A549 and H1650 cells were incubated in RPMI 1640 (Gibco, C11875500BT) supplemented with 10 % fetal bovine serum (FBS, 3022A, Umedium, Anhui, China) at 37 °C and 5 % CO_2_. The cells were passaged when they reached 90–95 % confluence.

### 2.3 Cell treatment

The LINC00323 overexpression plasmid (NR_024100.1, OE-LINC00323), AKAP1 overexpression plasmid (NM_001242902.1, OE-AKAP1), empty plasmid (OE-CTRL), shRNAs for the knockdown of LINC00323 (sh-LINC00323-1: 5′-UAUAUGAACUCUAUACAACCU-3’; sh-LINC00323-2: 5′-UCAAAACUGGUUUUUACUGUG-3′) and AKAP1 (sh-AKAP1-1: 5′-UGUUAAACACUAUCCUUUGGC-3’; sh-AKAP1-2: 5′-AGAAAGAAAGGACUUUCUGGA-3′), and sh-control (sh-CTRL) were purchased from Synbio Technologies Co., Ltd. (Suzhou, China). The cells were collected, counted, seeded in 6-well plates (1 × 10^5^ cells/well), and transfected with the overexpression plasmids or shRNAs via Lipofectamine 3000 (Invitrogen, Carlsbad, CA, USA) according to the manufacturer's instructions. LINC00323-silenced H1650 and A549 cells were also treated with 50 μg/mL cycloheximide (CHX, HY-12320, MedChemExpress, Shanghai, China) for 0, 4, 8, or 12 h or with 5 μM MG132 (HY-13259, MedChemExpress, Shanghai, China) for 8 h.

### 2.4 qRT‒PCR

Total RNA was extracted from each group of cells or milled tissues with TRIzol reagent. After the purity and concentration of the total RNA were examined, reverse transcription was conducted with a PrimeScript RT reagent kit (Takara, RR047A). LINC00323, VEGFA, and AKAP1 mRNA levels were examined with Fast SYBR Green Mix (Applied Biosystems) with cDNA as a template and primers (Table 1).

**Table 1:** Primer sequences for qRT-PCR.

Gene	Forward primer (5’→3′)	Reverse primer (5’→3′)
LINC00323	GGCTGACAGGAGGATGAGTG	GGTGCGAGTCTGACATTCCA
AKAP1	AATGTCTCTGTGTTCCACCC	CCATCCTGGAGGCTTGAAGT
VEGFA	ACGAAAGCGCAAGAAATCCC	CTCCAGGGCATTAGACAGCA
GAPDH	CTCCTCCTGTTCGACAGTCAGC	CCCAATACGACCAAATCCGTT

### 2.5 Western blot

Total protein was extracted from each group of cells and ground after lysis with precooled RIPA buffer (Beyotime, China). After quantification, 40 μg of protein was separated and transferred to PVDF membranes (Millipore). After 2 h of incubation in 5 % skim milk, the samples were incubated overnight at 4 °C with primary antibodies, including anti-VEGFA antibody (1:8000, Proteintech Cat# 19003-1-AP, RRID: AB_2212657), anti-AKAP1 antibody (1:1000, Proteintech Cat# 15618-1-AP, RRID: AB_2225484), anti-ubiquitin antibody (Proteintech Cat# 10201-2-AP, RRID: AB_671515) and anti-GAPDH antibody (1:20000, Proteintech Cat# 10494-1-AP, RRID: AB_2263076). After washing, the samples were incubated with a secondary antibody (1:5000, Proteintech Cat# SA00001-2, RRID: AB_2722564) for 1 h. After washing, the samples were titrated with enhanced chemiluminescence (ECL) reagent (GE Healthcare Life Sciences, UK) for development and imaging. The gray value of each band was analyzed using ImageJ software.

### 2.6 Immunohistochemistry (IHC)

Patient tissues were fixed, paraffin-embedded, and cut into 4-μm-thick tissue sections. After dewaxing and rehydration, the sections were processed with 3 % H_2_O_2_ for 10 min and placed in citrate buffer at microwave temperature for antigen repair. After being blocked with goat serum (Gibco, USA), the sections were treated with anti-VEGFA (1:400), anti-CD31 (1:3000, Proteintech Cat# 11265-1-AP, RRID: AB_2299349), or anti-AKAP1 (1:300) overnight at 4 °C and then with Multi-rAb Polymer HRP-Goat Anti-Rabbit Recombinant Secondary Antibody (H + L) (Proteintech Cat# RGAR011, RRID: AB_3094534) at 37 °C for 1 h. After being washed, the sections were stained with DAB, rinsed with tap water, counterstained with hematoxylin, differentiated with ethanol hydrochloride, dehydrated, cleared, and sealed with neutral gum. The protein expression was observed and photographed using a light microscope. The staining intensity and proportion of positive cells were scored.

### 2.7 Microvessel density (MVD) detection

MVD was examined using CD31/periodic acid-Schiff (PAS) double staining on the basis of previous research [26]. Briefly, the tissue sections were subjected to antigen retrieval via heat induction to expose the CD31 and PAS antigens in the tissue. Then, the sections were incubated with an anti-CD31 antibody, washed, and incubated with a biotinylated secondary antibody (Abcam). After washing, the sections were stained with a PAS staining solution to monitor polysaccharides. After washing, the distribution of CD31 and PAS was observed and recorded microscopically.

### 2.8 CCK-8

The treated cells (1 × 10^4^ cells/well) in 96-well plates were cultured for 0, 12, 24, and 48 h and treated with 10 μL of CCK-8. After 4 h of incubation, the absorbance was monitored at 450 nm using a microplate reader.

### 2.9 EdU staining

The transfected cells were inoculated in 24-well plates (1 × 10^4^ cells/well) and cultured at 37 °C for 12 h. The cells in each well were treated with 50 μM EdU for 2 h, 4 % paraformaldehyde for 30 min, 2 mg/mL glycine, and 0.5 % Triton X-100. Then, the cells were treated with Apollo at 37 °C for 30 min. After washing, the cells were stained with DAPI (Sigma) for 10 min. Images were acquired via fluorescence microscopy.

### 2.10 Transwell

The treated cell suspension (2 × 10^5^ cells/ml, 200 μL) and culture medium supplemented with 10 % FBS (600 μL) were added to the upper and lower chambers of the Transwell plate, respectively. After being cultured for 24 h, the excess cells were gently removed. The migrated cells were fixed and stained. After being washed with PBS, the migrated cells were observed and counted under a light microscope (BX51, Olympus Corporation).

### 2.11 Tube formation assay

Matrigel (0.5 mmol/L, BD Biosciences) was coated on a prechilled 96-well plate at 37 °C for 30 min. The cells were starved without serum, resuspended in DMEM to make a cell suspension, and seeded at 1 × 105 cells/mL into the 96-well plate. After 6–8 h, tube formation was observed under a microscope, and the closed tube structure network was imaged in three randomly selected areas under a microscope (Leica).

### 2.12 RNA immunoprecipitation (RIP) assay

A549 and H1650 cells were collected and fixed with formaldehyde. Then, the cells were resuspended for lysis with freshly prepared RIP lysis buffer on ice for 5 min. Protein A/G magnetic beads were prepared according to specific instructions. The lysates were incubated with 5 μg of AKAP1 antibody or IgG (ProteinTech Group) conjugated to protein A/G magnetic beads (Millipore) in 500 μl of IP buffer containing an RNase inhibitor (Thermo Fisher) overnight at 4 °C. IP complexes were treated with proteinase K (Thermo Fisher) at 52 °C for 1 h. After RNA was purified, the expression of LINC00323 was examined by qRT-PCR assay.

### 2.13 RNA pull-down

LINC00323 was transcribed with an RNA In Vitro Transcription Kit (Thermo Fisher Scientific, USA). Biotin labeling was performed using the Biotin RNA Labeling Mixture. Biotinylated RNA was incubated with streptavidin-conjugated magnetic beads overnight at 4 °C. The mixtures were collected by centrifugation of the magnetic beads after overnight incubation with lysates from groups of A549 and H1650 cells. After elution and denaturation, the potential binding proteins were identified using liquid chromatograph mass spectrometer/mass spectrometer (LC-MS/MS) by APTBIO (Shanghai, China), and the resulting RNA‒protein complexes were analyzed and confirmed via western blotting.

### 2.14 Immunoprecipitation (IP)

A Pierce Co-IP Kit (Pierce, 88804) was used to measure the level of AKAP1 ubiquitination. The cell lysates were mixed with AKAP1 antibody (1:200) at 4 °C overnight and then incubated for 1 h at 25 °C with prewashed A/G magnetic beads. The magnetic beads were washed with elution buffer at 25 °C and eluted with 2 × SDS buffer, followed by Western blot analysis.

### 2.15 Statistical analysis

The data are presented as the mean ± SD and were statistically analyzed using SPSS 21.0 software. The results were analyzed using a *t*-test or one-way ANOVA. Overall survival (OS) was analyzed via Kaplan‒Meier analysis on the basis of gene expression. Correlation analysis was conducted using Pearson analysis. *P* < 0.05 indicated statistical significance.

## 3 Results

### 3.1 LINC00323 is related to metastasis, poor prognosis, and MVD in NSCLC patients

**Fig. 1:**
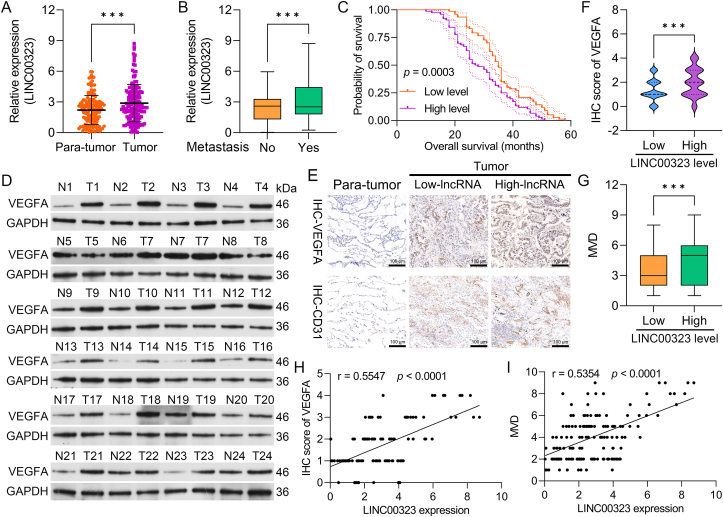
**LINC00323 is upregulated in NSCLC tissues and is related to metastasis, patient prognosis, VEGFA and CD31 expression, and MVD.** (A) LINC00323 expression was confirmed using qRT‒PCR in para-tumor and tumor (NSCLC) tissues. (B) qRT‒PCR analysis of LINC00323 expression in nonmetastatic and metastatic NSCLC patient tissues. (C) Survival analysis of NSCLC patients with high and low LINC00323 expression. (D) Western blotting was used to evaluate VEGFA expression in para-tumor (N) and NSCLC tissues (T). (E) IHC was used to monitor VEGFA and CD31 expression in NSCLC tissues from patients with high and low LINC00323 expression. (F) IHC score of VEGFA. (G) The MVD was assessed. Correlation analysis between LINC00323 expression and the IHC score of VEGFA (H) and the MVD (I). ∗∗∗*P* < 0.001.

To determine the relationship between LINC00323 and NSCLC, LINC00323 levels were examined in collected normal and NSCLC tissues. qRT‒PCR revealed that LINC00323 was upregulated in NSCLC tissues (Fig. 1A). LINC00323 levels were also greater in tissues from patients with metastatic NSCLC than in those from patients with nonmetastatic NSCLC (Fig. 1B). LINC00323 was categorized into high- and low-expression groups using the median level as the cutoff value. The data indicated that the upregulation of LINC00323 indicates a poor prognosis for NSCLC patients (Fig. 1C). In addition, the western blot results indicated that VEGFA expression was notably increased in NSCLC tissues (T) compared with para-tumor tissues (N) (Fig. 1D). According to the IHC results, VEGFA and CD31 expression was upregulated in NSCLC tissues from the LINC00323 high-expression group (Fig. 1E and F). The MVD was also greater in the LINC00323 high-expression group than in the LINC00323 low-expression group (Fig. 1G). The results revealed that the LINC00323 level was positively correlated with the VEGFA and MVD levels (Fig. 1H and I). Thus, LINC00323 plays a role in promoting the metastasis of NSCLC.

### 3.2 LINC00323 accelerates the proliferation and metastasis of NSCLC cells

**Fig. 2:**
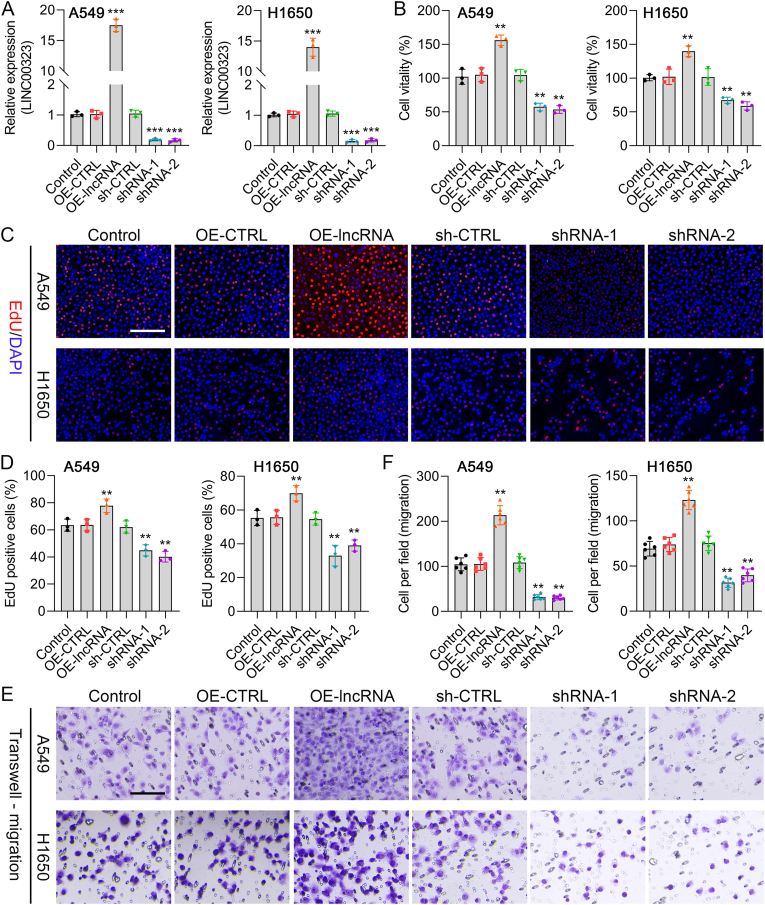
**The effect of LINC00323 on the proliferation and migration of A549 and H1650 cells.** A549 and H1650 cells were transfected with OE-LINC00323, sh-LINC00323-1, or sh-LINC00323-2. (A) LINC00323 expression was evaluated via qRT‒PCR. (B) CCK-8 assay for cell viability. (C) EdU staining revealed cell proliferation. (D) EdU-positive cells were counted. (E) Cell migration was confirmed by the Transwell assay. ∗∗*P* < 0.01, ∗∗∗*P* < 0.001.

By transfection, LINC00323 was first overexpressed and silenced in A549 and H1650 cells. As shown by the qRT‒PCR results, LINC00323 levels were elevated after transfection with the LINC00323 overexpression plasmid and prominently decreased after LINC00323 shRNA transfection in A549 and H1650 cells (Fig. 2A). According to the CCK-8 and EdU staining results, cell viability was clearly increased in the LINC00323 overexpression group and decreased in the LINC00323 silenced group (Fig. 2B–D and Fig. S1). Transwell assays also revealed that LINC00323 overexpression promoted NSCLC migration and that LINC00323 silencing inhibited NSCLC cell migration (Fig. 2E and F). Overall, aberrant expression of LINC00323 could alter the proliferation and metastasis of NSCLC cells.

### 3.3 LINC00323 enhances VM formation and upregulates VEGFA expression in NSCLC cells

**Fig. 3:**
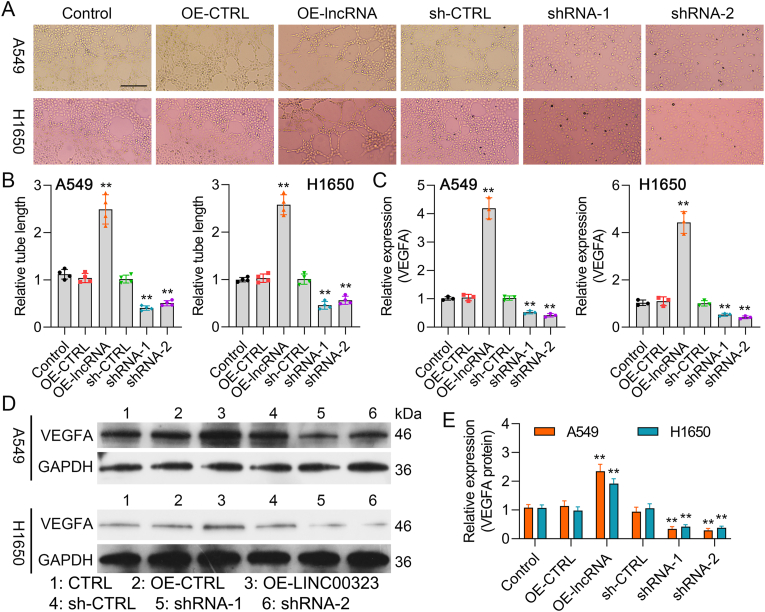
**The influence of LINC00323 on VM formation and VEGFA expression in A549 and H1650 cells.** (A) After LINC00323 overexpression or silencing, cell tube formation was examined using a tube formation assay. (B) Quantitative analysis of relative tube length. (C) qRT‒PCR was used to evaluate the regulatory effect of LINC00323 on VEGFA expression. (D) VEGFA expression was measured using Western blotting. (E) VEGFA levels were quantified. ∗∗*P* < 0.01.

Moreover, LINC00323 overexpression dramatically enhanced VM, and this effect was inhibited by LINC00323 silencing in NSCLC cells (Fig. 3Aand B). The qRT‒PCR results revealed that VEGFA expression was markedly greater in the LINC00323-overexpressing group than in the OE-CTRL group and lower in the LINC00323-silenced group than in the sh-CTRL group (Fig. 3C). Western blot results further confirmed the positive regulatory effect of LINC00323 on VEGFA expression (Fig. 3D and E). These data indicated that LINC00323 could lead to the enhancement of VM in NSCLC cells.

### 3.4 LINC00323 regulates the degradation of ubiquitinated AKAP1

**Fig. 4:**
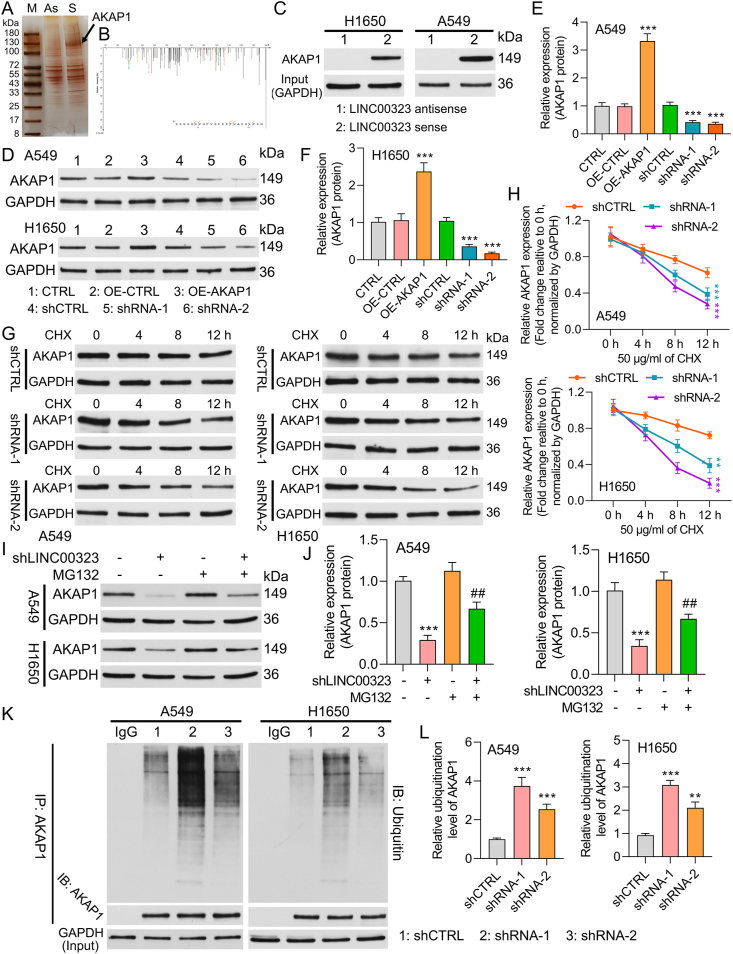
**LINC00323 interacts with AKAP1.** (A) RNA pull-down assays were performed using biotinylated sense or antisense LINC00323. Proteins from A549 cell extracts were separated via SDS-PAGE and visualized by silver staining. (B) AKAP1 was analyzed by LC-MS/MS. (C) RNA pull-down was applied to determine the interaction between LINC00323 and AKAP1. (D) The regulatory effect of LINC00323 on AKAP1 expression was verified by Western blotting. (E–F) The relative level of AKAP1 was quantified in line with the gray value. (G–H) After LINC00323 silencing, AKAP1 expression in H1650 and A549 cells treated with 50 μg/mL CHX was confirmed by Western blotting. (I–J) Western blotting was used to determine AKAP1 expression in LINC00323-silenced H1650 and A549 cells treated with MG132 for 4 h. (K) The ubiquitination level of the AKAP1 protein was verified through IP, and (L) the AKAP1 protein level was quantitatively analyzed. ∗∗*P* < 0.01, ∗∗∗*P* < 0.001; ##*P* < 0.01 vs. the sh-LINC00323 group.

To explore the target genes related to angiogenesis that may be regulated by LINC00323, potential functional proteins that interact with LINC00323, were identified via LC-MS/MS. This study identified 783 potential regulatory proteins (Supplementary Table 1) with AKAP1 on the top, which basing on the label-free quantification (LFQ) intensity and intensity-based absolute quantification (iBAQ). Silver staining and mass spectrometry analyses identified AKAP1 as a protein that interacts with LINC00323 (Fig. 4A–B). IHC data revealed that AKAP1 was upregulated in NSCLC tissues (Supplementary Figs. 2A and 2B). LINC00323 and AKAP1 expression were also positively correlated in NSCLC tissues (Supplementary Fig. 2C), and upregulation of AKAP1 protein indicated a poor prognosis for NSCLC patients (Supplementary Fig. 2D). The RIP results showed that the level of LINC00323 was markedly enriched in the anti-AKAP1 group, suggesting that LINC00323 could bind to AKAP1 protein (Supplementary Fig. 3). An RNA pull-down assay also verified the interaction between LINC00323 and AKAP1 (Fig. 4C). According to the Western blot results, LINC00323 overexpression upregulated AKAP1, and LINC00323 silencing downregulated AKAP1 in NSCLC cells (Fig. 4D–F). Next, 50 μg/mL CHX was added to NSCLC cells in the sh-CTRL and shLINC00323 groups. Western blot analysis revealed that CHX treatment prominently reduced the decrease in the protein level of AKAP1 caused by LINC00323 knockdown (Fig. 4G–H). The reduction in the level of AKAP1 in the LINC00323-silenced group was dramatically reversed after MG132 intervention for 4 h, further indicating that knockdown of LINC00323 promoted AKAP1 protein degradation (Fig. 4I–J). In addition, the IP results indicated that LINC00323 knockdown increased the ubiquitination level of the AKAP1 protein, suggesting that LINC00323 knockdown enhances the ubiquitination of the AKAP1 protein (Fig. 4K–L). These results suggested that LINC00323 could target AKAP1 and reduce the degree of ubiquitination of the AKAP1 protein.

### 3.5 AKAP1 knockdown prevents NSCLC cell malignant processes

**Fig. 5:**
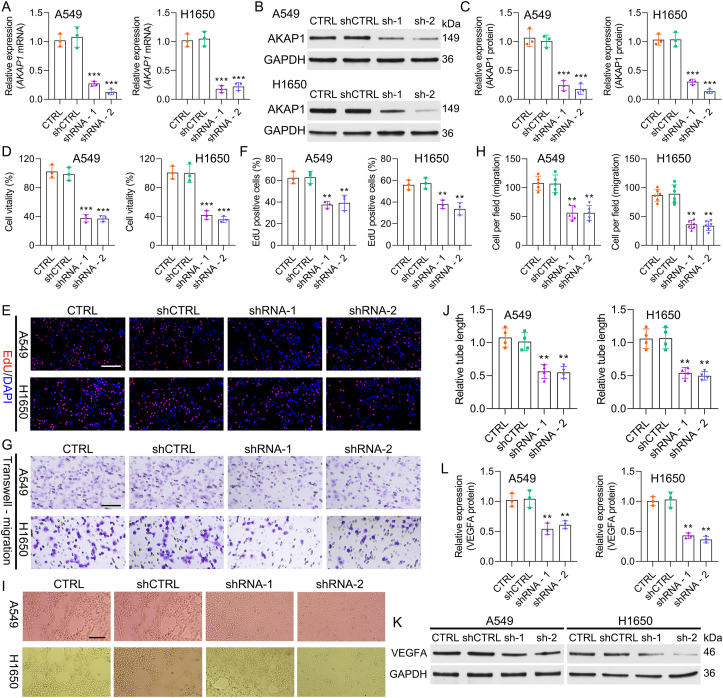
**AKAP1 knockdown prevents NSCLC cell malignant processes.** H1650 and A549 cells were transfected with sh-AKAP1-1 or sh-AKAP1-2. (A) qRT‒PCR was conducted to evaluate the mRNA level of AKAP1. (B–C) Western blot analysis demonstrated the change in AKAP1 expression. (D) Cell viability was determined by the CCK-8 assay. (E) Cell proliferation was assessed using an EdU assay. (F) The number of EdU-positive cells was calculated. (G–H) Transwell assays were used to assess changes in cell migration. (I–J) A tube formation assay was applied to detect cell tube formation. (K–L) Western blot analysis of VEGFA. ∗∗*P* < 0.01, ∗∗∗*P* < 0.001.

Furthermore, the function of AKAP1 was explored in NSCLC cells. sh-AKAP1-1 and sh-AKAP1-2 were transfected into H1650 and A549 cells. By validation, we found that AKAP1 expression was notably reduced in the AKAP1-silenced group, indicating successful knockdown of AKAP1 (Fig. 5A–C). NSCLC cell proliferation was also prominently reduced after AKAP1 knockdown (Fig. 5D–F and Fig. S4). AKAP1 knockdown had a suppressive effect on NSCLC cell migration (Fig. 5G–H). AKAP1 knockdown also dramatically decreased VM in NSCLC cells (Fig. 5I–J). In addition, AKAP1 knockdown markedly downregulated VEGFA protein expression in NSCLC cells (Fig. 5K– L). Taken together, these findings indicate that AKAP1 knockdown plays an oncogenic role in NSCLC progression.

AKAP1 overexpression partly reverses the attenuating effects of LINC00323 silencing on the proliferation and VM of NSCLC cells.

**Fig. 6:**
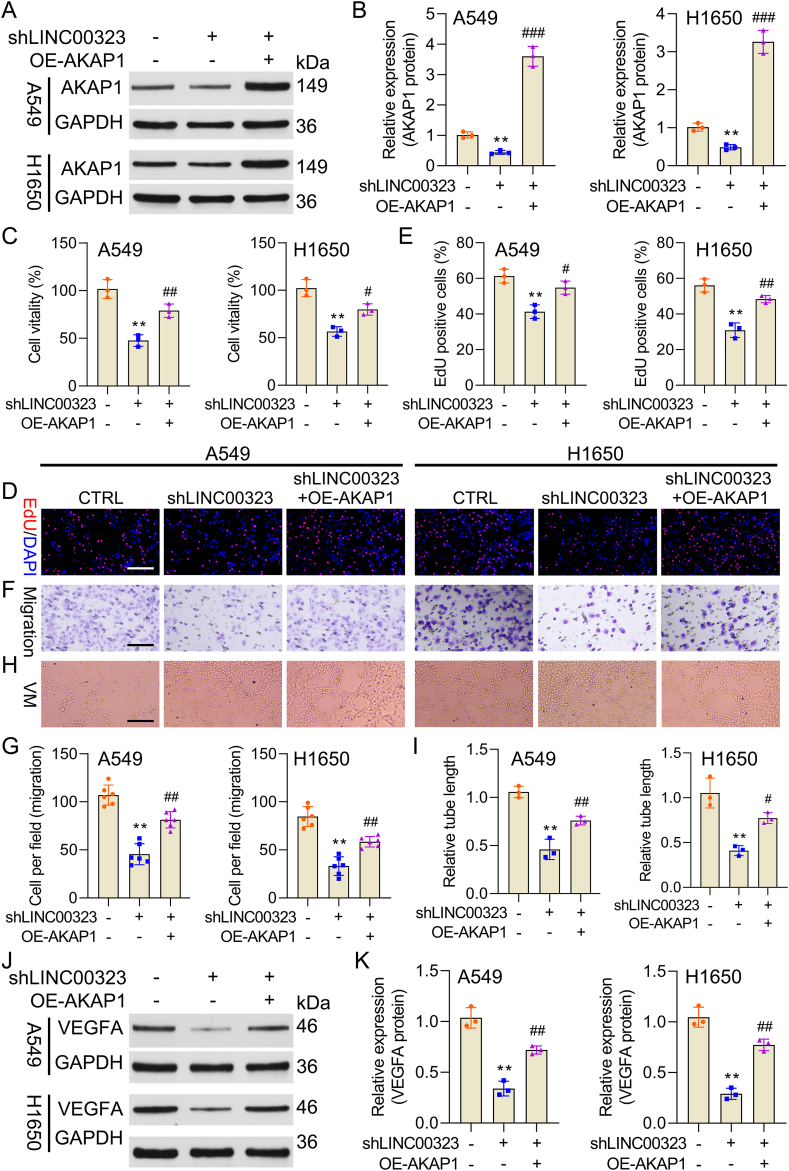
**AKAP1 overexpression attenuates the suppressive effect of LINC00323 silencing on NSCLC cell proliferation, migration, and VM.** sh-LINC00323 and OE-AKAP1 were cotransfected into H1650 and A549 cells. (A) The protein level of AKAP1 was assessed by western blot. (B) AKAP1 expression was quantitatively assessed. (C) CCK-8 assay for the examination of cell viability. (D) An EdU assay revealed a change in cell proliferation. (E) The number of EdU-positive cells was computed. (F) Transwell assays were used to assess changes in cell migration. (G) The number of migrated cells per field was calculated. (H) A tube formation assay. (I) The relative tube length was calculated. (J) Western blot analysis of VEGEA expression. (K) VEGEA expression levels were quantified. ∗∗*P* < 0.01 vs. the control group; #*P* < 0.05, ##*P* < 0.01, ###*P* < 0.001 vs. the sh-LINC00323 group.

Importantly, the present study used rescue experiments to confirm whether LINC00323 can influence malignant cellular progression by regulating AKAP1 in NSCLC. H1650 and A549 cells were cotransfected with sh-LINC00323 and OE-AKAP1. Western blot results revealed that AKAP1 overexpression markedly upregulated AKAP1 expression mediated by LINC00323 silencing in NSCLC cells (Fig. 6A and B). Moreover, AKAP1 overexpression dramatically reversed the reduction in NSCLC cell proliferation, migration, and VM induced by LINC00323 silencing (Fig. 6C–I). Moreover, the reduction in VEGEA expression caused by LINC00323 silencing could also be partially reversed by AKAP1 overexpression in NSCLC cells (Fig. 6J and K). In summary, LINC00323 silencing suppresses NSCLC cell proliferation and VM formation by downregulating AKAP1.

## 4 Discussion

NSCLC metastasis is a complex multigene process [27]. Studies have shown that aberrantly expressed lncRNAs can accelerate or prevent various cancer processes, including NSCLC [28,29]. For example, LINC01123 accelerates aerobic glycolysis and proliferation in NSCLC cells via the miR-199a-5p/c-Myc axis [30]. LINC02159 enhances NSCLC progression via ALYREF/YAP1 [31]. The lncRNA MT1JP prevents NSCLC progression by activating the PTEN/AKT pathway [32]. The lncRNA XIST is oncogenic in colorectal, bladder, and gastric cancers and plays a tumor-suppressor role in breast and prostate cancers [[33], [34], [35], [36]]. Therefore, it is vital to study the role of lncRNAs in different types of tumors. LINC00323 is a relatively newly discovered lncRNA that has rarely been studied, and its role in NSCLC has not yet been reported. A recent study revealed that LINC00323 acts as a procarcinogenic agent in advanced gastric cancer, and its high expression is correlated with poor patient prognosis [25]. In addition, LINC00323 can alleviate diabetic nephropathy by affecting M1 macrophage polarization [37]. These studies confirmed that LINC00323 significantly accelerates disease progression. This study revealed for the first time that LINC00323 is highly expressed in NSCLC tissues and is related to metastasis and poor patient prognosis.

VM is a fluid-mediated, extracellular matrix-rich tubular network formed by tumor cells [11]. VM can effectively complement endothelium-dependent vascular structures to provide an adequate blood supply for tumor growth [20]. Double staining with CD34 and PAS is a classic method for identifying tumor vascular structures and VM [38]. CD34 and PAS staining are frequently applied to identify endothelial structures in tumor tissue and basement membrane structures in the tumor vasculature, respectively. VM is characterized by negative CD34 and positive PAS staining [39]. In this study, CD31 staining was applied to identify VM in NSCLC tissues. The results revealed that LINC00323 was associated with MVD in NSCLC patients. In addition, LINC00323 expression was correlated with VEGFA expression. VEGFA is a powerful angiogenic factor that strongly induces endothelial cell proliferation and tubular structure formation [40]. VEGFA and VM are correlated. VM is reportedly associated with various malignant biological behaviors in tumors [6,10]. Previously studies have shown that LINC00323 overexpression might serve as a novel independent prognostic factor for survival of gastric cancer patients [25], and LINC00323 expression was associated with osteosarcoma recurrence [41], and that LINC00323 involved in the progression of breast cancer via participating in the glycosphingolipid biosynthesis pathway and the regulation of transcription and mast cell activation biological processes [42]. However, no previous study has reported the role of LINC00323 in VM in tumors. In the present study, we found that silencing LINC00323 markedly decreased the proliferation, migration, and VM of NSCLC cells, while overexpressing LINC00323 accelerated these biological processes in NSCLC cells. These findings suggest that LINC00323 may regulate VM in NSCLC cells.

LncRNAs can regulate the post-translational modifications by binding to the proteins directly [43,44]. A recent study revealed that LINC00987 is as a potential tumor suppressor gene in tumorigenesis, operating through its binding with SND1 to facilitate phosphorylation and subsequent degradation [45]. To investigate the possible mechanisms by which LINC00323 affects NSCLC cell VM, this study screened potential proteins that interact with LINC00323 by LC-MS/MS. Through comprehensive analysis, we found that AKAP1, an angiogenesis-related gene, may be a regulatory target gene of LINC00323. AKAP1 is the first discovered scaffold protein in the A-kinase anchoring protein (AKAP) family and can recruit protein kinase A (PKA) and other proteins and mRNAs to the mitochondrial outer membrane, thereby affecting mitochondrial function and related pathological processes [46]. However, no detailed studies on the specific mechanisms by which AKAP1 directly affects VM have been reported. However, considering the role of AKAP1 in regulating mitochondrial function and cardiovascular physiological processes [47], it may indirectly affect the formation and function of blood vessels, including VM, by affecting mitochondrial energy metabolism and signal transmission. Our results proved that AKAP1 was upregulated and positively correlated with LINC00323 in NSCLC tissues. Functionally, AKAP1 knockdown prevented the proliferation, migration, and VM formation of NSCLC cells. Moreover, LINC00323 can interact with AKAP1. Moreover, we found that the knockdown of LINC00323 enhances the ubiquitination of AKAP1, suggesting that the regulation of AKAP1 by LINC00323 may be mediated by its ubiquitination. We also verified that the attenuating effect of LINC00323 silencing on cell proliferation, migration, and VM is achieved by regulating AKAP1 in NSCLC cells. However, the study has shortcomings, such as *in vivo* experimental validation.

## 5 Conclusions

In summary, silencing LINC00323 attenuated NSCLC cell proliferation, migration, and VM, and this mechanism may be related to the ubiquitination of AKAP1. Therefore, LINC00323 may be a potential target for NSCLC treatment.

## CRediT authorship contribution statement

**Bin Ke:** Writing – review & editing, Visualization, Methodology, Investigation, Formal analysis, Data curation. **Hai Zhong:** Writing – review & editing, Methodology, Data curation. **Yuxin Gong:** Writing – review & editing, Methodology, Formal analysis. **Xiaofei Chen:** Writing – review & editing, Investigation. **Chenxin Yan:** Writing – review & editing, Investigation. **Lin Shi:** Writing – review & editing, Writing – original draft, Visualization, Project administration, Funding acquisition, Conceptualization.

## Ethics approval and consent to participate

The study was approved by The Ethics Committee of Zhujiang Hospital of Southern Medical University (Approval number: 2024-KY-105-01). All the subjects signed an informed consent form.

## Availability of data and materials

The datasets generated during and/or analyzed during the current study are available from the corresponding author on reasonable request.

## Funding

The research is supported by grants from 10.13039/501100001809National Natural Science Foundation of China (82074159), 10.13039/501100021171Basic and Applied Basic Research Foundation of Guangdong Province (2021A1515011611) and Medical Science and Technology Research Fund of Guangdong Province (C2022062).

## Declaration of competing interest

The authors declare that they have no known competing financial interests or personal relationships that could have appeared to influence the work reported in this paper.
