# Supplementary material for: LINC00323 knockdown suppresses the proliferation, migration, and vascular mimicry of non-small cell lung cancer cells by promoting ubiquitinated degradation of AKAP1
Source: Noncoding RNA Res. 2024 Dec 14;11:131–40. doi: 10.1016/j.ncrna.2024.12.006 (PMC11720444; doi:10.1016/j.ncrna.2024.12.006)
Supplement: Multimedia component 1 [file mmc1.docx]

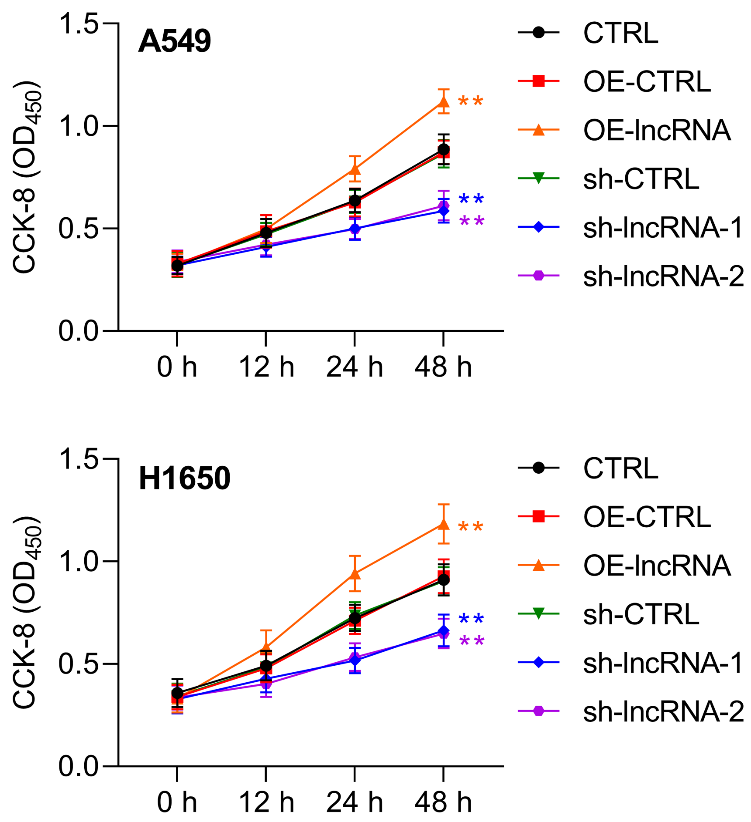


**Supplementary Figure 1. A549 and H1650 cells were transfected with LINC00323 overexpression plasmids and** **shRNA expression vectors, CCK-8 assay was performed to detect cell proliferation at 0, 12, 24, and 48 h.**


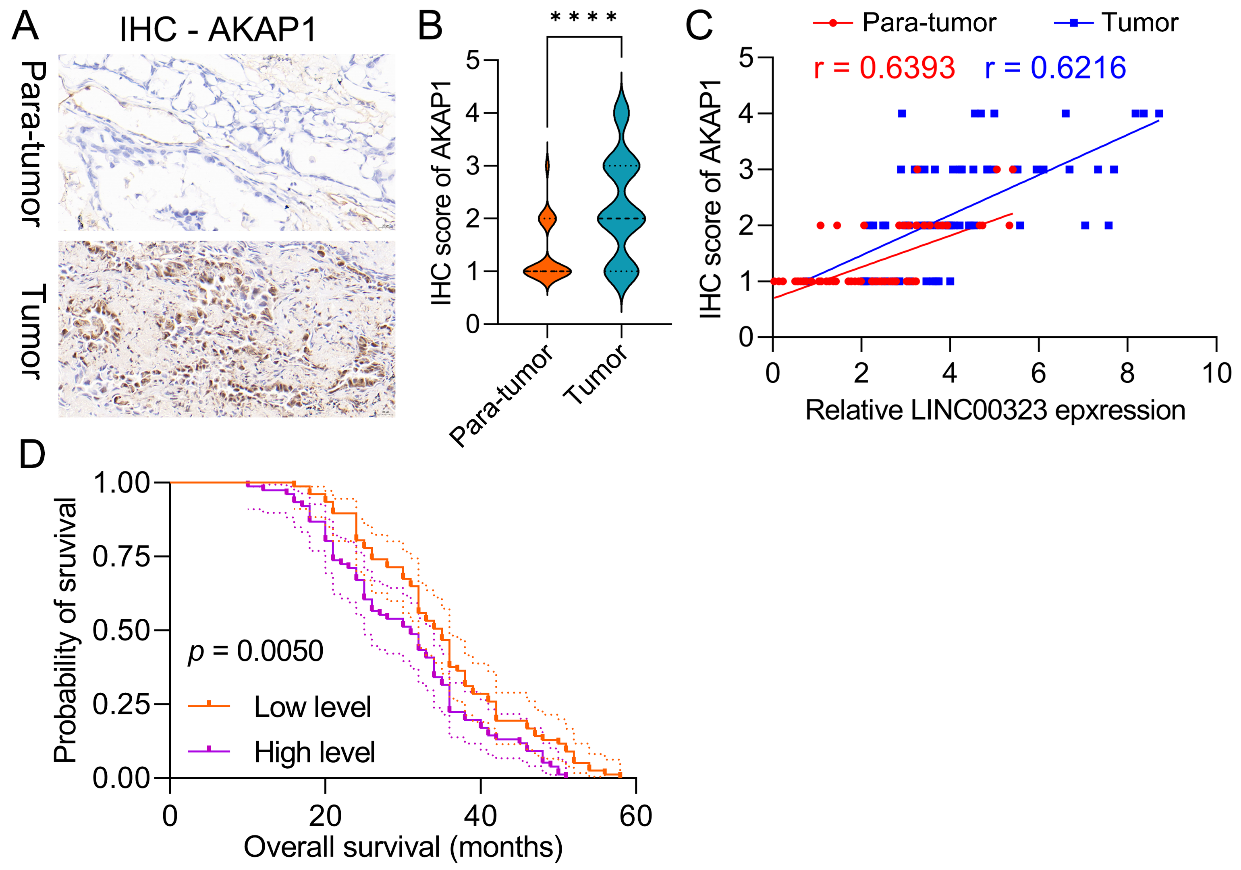


**Supplementary Figure 2. Analysis of AKAP1 expression and its correlation with LINC00323 expression in NSCLC tissues.** (A) IHC analysis of AKAP1 expression. (B) The IHC score of AKAP1. (C) Correlation analysis between the IHC score of AKAP1 and the expression of LINC00323 in normal and NSCLC tissues. (D) Survival analysis of NSCLC patients with high and low AKAP1 expression. *****P*<0.0001.


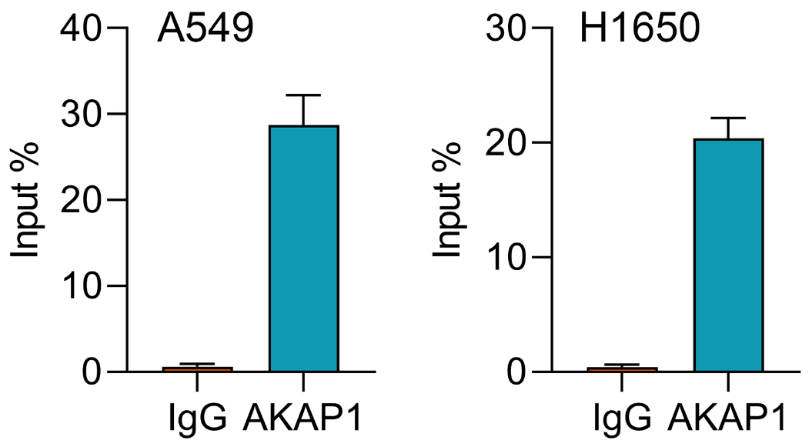


**Supplementary Figure 3. Verification of the interaction between LINC00323 and AKAP1.** Interaction between AKAP1 protein and LINC00323 was evaluated using RIP assay in A549 and H1650 cells.


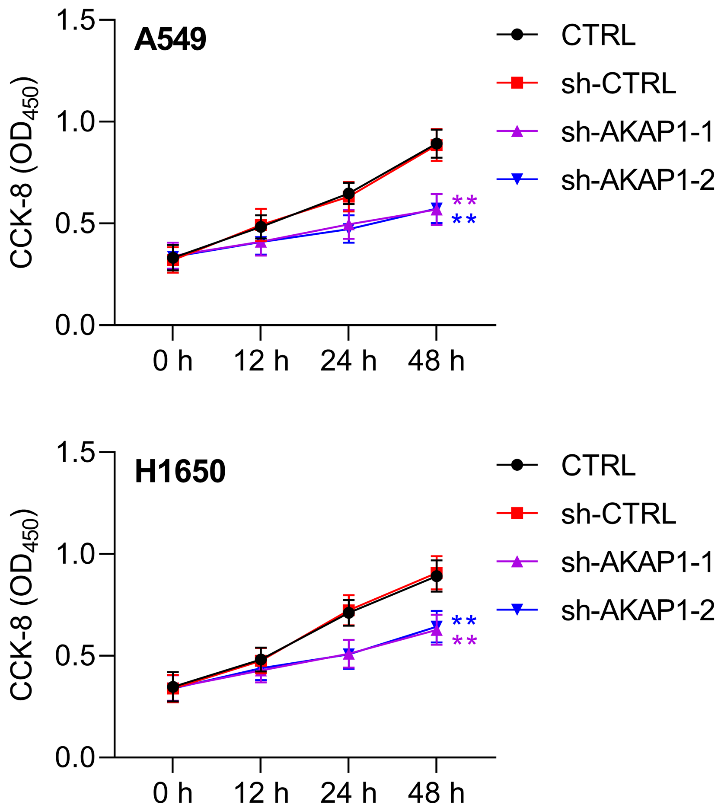


**Supplementary Figure 4. A549 and H1650 cells were transfected with AKAP1** **shRNA expression vectors, CCK-8 assay was performed to detect cell proliferation at 0, 12, 24, and 48 h.**
